# A review of web-based support systems for students in higher education

**DOI:** 10.1186/s13033-017-0165-z

**Published:** 2017-09-25

**Authors:** Marietta Papadatou-Pastou, Rhianna Goozee, Erika Payne, Alexia Barrable, Patapia Tzotzoli

**Affiliations:** 1 School of Education, Research Centre for Psychophysiology and Education, National and Kapodistrian University of Athens, Athens, Greece; 2 Independent Scholar, London, UK; 3 University of Derby, Kedleston Rd, Derby, UK; 4 University of Dundee, Nethergate, Dundee, Scotland UK; 5 iConcipio Ltd, London, UK

**Keywords:** Online support, Online interventions, Students, Mental health, Higher education

## Abstract

**Background:**

Recent evidence suggests that there is an increasing need for accessible and anonymous services to support higher education (HE) students suffering from psychological and/or academic difficulties. Such difficulties can lead to several negative outcomes, including poor academic performance, sub-optimal mental health, reduced study satisfaction, and dropout from study. Currently, universities in the UK lack financial resources and the on-campus mental health services traditionally offered to students are increasingly economically unsustainable. Compounded by the perceived stigma of using such services, mental health providers have been driven to address the escalating needs of students through online services.

**Methods:**

In this paper, we review online support systems identified through a literature search and a manual search of references in the identified papers. Further systems were identified through web searches, and systems still in development were identified by consultation with researchers in the field. We accessed systems online to extract relevant information, regarding the main difficulties addressed by the systems, the psychological techniques used and any relevant research evidence to support their effectiveness.

**Conclusion:**

A large number of web-based support systems have been developed to support mental health and wellbeing, although few specifically target HE students. Further research is necessary to establish the effectiveness of such interventions in providing a cost-effective alternative to face-to-face therapy, particularly in certain settings such as HE institutions.

## Background

Recently, there has been growing interest in the wellbeing of students in higher education (HE) [1]. This reflects the fact that many students face both mental health difficulties [2, 3] and study skill difficulties [4, 5]. These difficulties likely result from the transition into HE, which is accompanied by financial burdens, moving away from friends and family to live independently, lifestyle changes, and academic worries [6]. Consequently, many students are struggling with psychological problems, such as moderate anxiety, depression, and stress [7]. Indeed, some experience more serious levels of anxiety and depression [8, 9], as well as eating disorders, sleep disorders or substance abuse [10]. Furthermore, the opportunity to go to university is now available to a wider section of society, meaning that people with mental health difficulties are more likely to enter HE than before [11]. In addition to psychological problems, almost all HE students report problems with study skills, an additional stressor of academic life [3].

The importance of psychological wellbeing among HE students has been highlighted by recent research showing that students with poor mental health experience less social contact with their fellow students and faculty, reduced study satisfaction, poor academic achievement, and lower graduation rates [12]. Furthermore, insufficient treatment of potentially recurrent disorders, such as depression, can have delayed negative consequences [13]. In one study, students described as having moderate or languishing mental health were more likely to report impaired academic performance than those free of mental disorder [14]. Moreover, unhealthy lifestyles and risk behaviours, such as substance or alcohol abuse, have been linked to psychological and academic difficulties [15]. The above may be linked with the fact that more than 8% of undergraduate students in UK HE institutions drop out in their first year [4] and more than one in five students fail to complete their studies [16].

Despite the problems they face, students may be reluctant to seek help. Several barriers to help-seeking have been proposed, including perceived stigma, accessibility (e.g., time or cost) and lack of knowledge about what services are available [17]. Some may be concerned that their problems will not be understood [18] or simply underestimate the impact their psychological wellbeing has on their academic success [13]. Young people also often rely on self-taught skills to manage their mental health [1]. It is likely that stigma may play a particularly prominent role in failure to seek help among young adults. For example, in a survey of 765 HE students diagnosed with a mental health condition, stigma was identified as the number one reason not to seek mental health services on campus [18].

Although stigma remains an obstacle to seeking help for mental health issues, there are signs that mental illness is now more often disclosed by HE students [19]. It has been suggested that increased discussion of mental health, reflected in the increased number of students disclosing a mental illness before they arrive at university, is a result of a more open society and more reliable early diagnosis [19]. Whilst increased openness is positive, these changes have placed greater demand on HE institutions to meet the increasing (and increasingly disclosed) needs of their student populations. This issue is further compounded by austerity measures and budget cuts for statutory services, implemented within a context of national financial hardship [11]. Given these challenges, the sustainability of traditional support services should be questioned and viable alternatives need to be explored.

One such alternative is web-based support, which recognizes the significant role that electronic media play in university students’ lives. Indeed, the Royal College of Psychiatrists [3] has proposed the use of web-based support, such as interactive Computerized Cognitive Behavioural Therapy (CCBT), for mental health. Such support is cost-effective, time saving, and highly accessible for HE students. Students may also prefer computerized self-help as a means to increase independence and self-reliance [20], and perceive it as less stigmatizing than traditional therapy [21]. Recipients of CCBT show reduced perceived stigma related to mental disorders [22]. Encouragingly, increasing evidence supports the efficacy of web-based interventions for mental health disorders [1, 23]. Nonetheless, there are still significant gaps in our knowledge of whether such technology will be effective in treating mental disorders in the HE context [21].

Recent years have witnessed a growth in web-based interventions for wellbeing, in addition to clinically diagnosed mental disorders [23]. There remain few that have been developed specifically for HE students, although some are now available. As well as targeting different audiences, systems offering online support for mental health are varied in the type and extent of support they offer. Some online interventions may involve face-to-face therapy, but this is not common to all systems. Such online therapy can be accessed via interactive computer interfaces on a personal computer or mobile device. In addition to offering therapy, some systems are used by patients and relatives to seek information on mental health and to research therapy options [24]. They can provide self-help strategies and tools to track wellbeing in the form of questionnaires and self-assessment surveys. Many offer online support groups and forums, where privacy and anonymity are preserved. Some systems that offer CCBT require referral from a medical professional, especially in the United Kingdom. Others allow self-referral, offering a detailed online assessment of mental health before use.

Online interventions are becoming increasingly prevalent in the HE context, but to our knowledge there has been no comprehensive review of online support systems that are currently available or in development that could support HE students. In this review, we systematically present internet-based mental health support systems. We aim to provide an overview of the systems, including the target populations, the psychological approach taken, and what research, if any, has been published regarding the effectiveness of the intervention, as well as related costs.

## Methods

We reviewed online support systems by identifying published literature, using a database search of PubMed with the keywords “online support”, “web support”, “CCBT”, and related terms, such as “online CBT” and “web-based support”. We also conducted a manual search of the reference list of identified papers. As there are some systems that have not been subjected to empirical research and so cannot be found in the published literature, we also identified further systems through web searches and consultation with researchers and clinicians in the field. We included any system designed to support psychological difficulties (mild to severe psychological symptoms). We did not restrict the search to systems designed specifically for HE students, but included any system that HE students can currently access or that they will be able to access following their development (i.e., we also included those designed for the general public). Where no publications were available, we obtained information about systems from their webpages, and where required from the team developing the system through direct communication. The search was completed in September 2016.

We accessed systems online to extract and review relevant information, including the country in which the system is available, the target group, the main difficulties addressed by the system, the psychological techniques used, how the system is accessed, and any relevant research evidence to support their effectiveness (Table 1). The evidence to support the development and use of the systems was outlined and rated according to the Oxford Centre for Evidence-Based Medicine 2011 Levels of Evidence [25] in a separate table (Table 2). This table aims to present a clear picture of the evidence base relating to the treatment benefits of each intervention, as well as to potentially highlight areas where more research might be needed.

**Table 1:** Key characteristics of identified web-based support systems

System	Country available	Target group	Psychological difficulties addressed	Psychological technique/intervention used	Additional features	Funding	Mode of delivery	Available as an app	Availability to UK HE students
Systems that were designed for use by HE students									
CALM	Ireland	HE students	Depression, anxiety, stress, insomnia, substance abuse and study skills	CBT Mindfulness	(a) Insomnia relief: sleep hygiene and stimulus control techniques (b) Drink and drug wise: tools to help with substance abuse (c) Podcasts covering topics such as diet and exercise	Cost to be covered by HE institution	Interactive multimedia—series of ebooks and colourful worksheets	No	Currently only available at certain HE institutions
MePlusMe	UK	HE students	Anxiety, stress, depression, low mood, general wellbeing, study skills	CBT Mindfulness	Self-directed skills training via personalized packages of techniques in 2D animated videos	To be confirmed	Multimedia, video, audio, and text	No	Not currently available
Mindful Kiwi	UK	HE students	Stress, health and wellbeing, study skills, and relationships	Mindfulness	8 weeks of therapy sessions (online or face to face)	Free	Topic focused guided meditations and group discussions	No	Not currently available
PLUS	UK	HE students	Depression, anxiety, self-esteem, perfectionism, substance abuse, eating disorders	CBT	Focused modules with text-based exercises	To be confirmed	Text-based modules with photographs and illustrations	No	Not currently available
Students Against Depression	UK	HE students	Depression, anxiety and suicide	CBT	(a) On campus peer-support programs (b) Blogging	Free	Non-linear text-based modules	No	Currently only available at certain HE institutions
theDesk	Australia	HE students	Stress, study and time management problems, mental and physical wellbeing	Mindfulness Relaxation	Relationship and health issues	Free	Multimedia modules with video and text	No	Available—registration needed
Systems that were designed for use by general population or other groups									
Beating the Blues	UK	General public	Depression and anxiety	CBT	8 weeks of online therapy sessions	Requires GP referral	Text-based information and homework	No	Available through primary care physician referral or private subscription
Big White Wall	UK	General public	Depression, anxiety, and stress	CBT	(a) Advice about abuse, bullying, relationships and lifestyle (b) Peer group support	Free via NHS referral or paid subscription	Text-based information, and community support	Yes	Available through primary care physician referral or private subscription
e-couch	Australia	General public	Depression, generalised anxiety/worry, social anxiety, relationship breakdown, and loss/grief	CBT IPT	(a) Advice on relaxation and physical activities (b) Self-help toolkit (c) Research platform (d) Internet support groups	Free	Free choice between 5 text-based modules	No	Available
FearFighter™	UK	General public	Panic and phobia	CBT	9 weeks of online therapy sessions	Packages available, starting at £129	9 linear sessions, text-based exercises	Yes	Available through paid subscription
Living life to the full	UK	General public	Depression, anxiety and stress	CBT	(a) 5 weeks of online sessions with recommended self-help books (b) Moderated forum	Free	Text-based sessions, audio and small group discussions	No	Available
MoodGym	Australia	General public, mainly aimed at young people	Depression and anxiety	CBT	Assertiveness and inter-personal skills training	Free	Linear sequence of 5 modules, text-based sessions	No	Available
NHS Silvercloud	UK	General public	Depression, anxiety, stress and eating disorders	CBT Mindfulness	(a) Interactive journaling (b) Mood and lifestyle charting (c) Social community (d) Dedicated supporter to monitor progress, and provide guidance	Free via NHS referral	Multimedia interactive modules (text, video and audio clips)	Yes	Available through primary care physician referral
ReachOut.com	Australia Ireland USA	Young people (15–25 years old)	Depression, anxiety, suicide, self-harm, substance abuse and study skills	CBT	(a) LGBTQ issues (b) Self-help tool (c) Moderated forum	Free	Text-based information, videos and community engagement	Yes	Available
Superbetter.com	USA	General public	Physical, social and mental wellbeing	Positive psychology Behaviour change	Physical and social skills (starting from basics, such as drinking more water)	Free	Video game, modules unlocked by completion of tasks within the game	Yes	Available
UBalancer	Australia	General public	Anxiety, stress, feeling overwhelmed, study and time management	Coaching Mindfulness	Sleeping pattern improvement	Subscription required	Structured coaching telephone calls, plus text-based supporting information	No	Not currently available

*CBT* Cognitive Behavioural Therapy, *CCBT* computerised cognitive behavioural therapy, *IPT* interpersonal therapy, *LGBTQ* lesbian, gay, bisexual, transgender and queer, *RCT* randomised controlled trial

**Table 2:** Levels of evidence available for web-based support systems

	System	Development studies	Effectiveness studies	Oxford CEBM level
Systems designed for HE students	CALM	None	None to date	Level 5
	MePlusMe	Development survey (Goozee et al. submitted) Proof of concept [25]	Feasibility study planned (September 2017)	Level 5
	Mindful Kiwi	None	Non-randomised pilot [28]	Level 3
	PLUS	None	Large RCT (*n* = 1047) [10]	Level 2
	Students Against Depression	None	None to date	Level 5
	theDesk	None	None to date	Level 5
Systems designed for the general public or other groups	Beating the Blues	Development and beta-testing study [28]	Efficacy in various groups and cost-effectiveness studies [29–37]	Level 2
	Big White Wall	None	Independent review [38] Cost-effectiveness study [39] Case-study [40]	Level 4
	e-couch	None	Small RCT (*n* = 21) [43] Effectiveness studies in various groups [44–46] Users’ perspectives [47, 48] Randomised double-blind (*n* = 5620) [49]	Level 2
	FearFighter™	None	Pilot study [50] Feasibility study [51] Efficacy study [52] RCT (*n* = 93) [53] Cost-effectiveness study [54] Feasibility study [55] RCT (*n* = 35) [56]	Level 3
	Living life to the full	None	None	Level 5
	MoodGym	None	Efficacy in various groups [57–61] Patient feedback [62] RCTs with positive results [58–63] RCTs showing no benefit over psychoeducation or usual care [64–66] RCT studying acceptability (*n* = 106) [68] Meta-analysis [69] Adherence studies [71–73]	Level 3
	NHS Silvercloud	None	Observational studies [74, 75] RCT effectiveness (*n* = 188) [76] RCT satisfaction (*n* = 281) [77]	Level 2
	ReachOut.com	None	User surveys [80, 81] Case-study [79]	Level 4
	Superbetter.com	None	Observational Study [83]	Level 4
	UBalancer	None	None	Level 5

## Results

### Identified systems

Our search yielded 18 web-based support systems (Table 1). Of these, nine were designed for use by the general public, seven were designed for HE students, one was designed for young people aged between 15 and 25 years, and one was designed for students and staff at a specific HE institution (University College Belfast). The majority of systems provided support for depression (*n* = 13) and anxiety (*n* = 12), with a large number also targeting generic symptoms of stress (*n* = 7). Other mental health issues targeted included eating disorders and suicide. Five of the systems specifically provided study skills training. Most systems were freely available (*n* = 8) but some required a referral from a healthcare professional.

The interventions offered by the systems were founded in a wide range of psychological therapies, with the majority stating that they offer CBT (*n* = 13) or CCBT (*n* = 11) techniques. Other techniques included mindfulness and Mindfulness Based Stress Reduction (MBSR) (*n* = 5), visualisation techniques (*n* = 2), relaxation techniques (*n* = 2), positive psychology (*n* = 2), and peer support (*n* = 2).

### Detailed descriptions of systems

#### (i) Systems designed for HE students

In this first section, we describe systems that have been specifically designed for HE students. The characteristics of each system are presented, while also addressing the level of evidence provided for each system to date.

- *CALM *(http://www.ucc.ie/en/wellbeing/calm/) Computer Aided Lifestyle Management (CALM) is an online multimedia system using interactive self-help tools to educate users about issues such as anxiety, depression, insomnia, stress, and substance misuse. The system was originally introduced at University College Cork (UCC), but is now available at other UK HEIs, including UCL and Newcastle University. The system comprises five programmes dealing with depression, anxiety, stress, sleep disorders, and substance abuse. Within each programme, there is an assessment unit to identify and track symptoms, a personal programme using CCBT, relaxation, mindfulness, and visualization techniques, and a psychoeducational unit. Although in use at several HEIs, there is currently no research published about the effectiveness of this system.
- *MePlusMe *(http://www.meplusme.com *—not yet live online*) MePlusMe is an online support system currently being developed by iConcipio Ltd. of which the authors of this paper form part of the research group. It is designed to support mild to moderate mental health difficulties (as shown by presenting symptoms of anxiety and depression) and study skills in HE students. Students with more severe difficulties are referred to the appropriate services for more intensive support via several filters throughout the system, including a “panic button”. A board of academics in UK HEIs and NHS clinicians oversaw the development of the system, ensuring it met the needs of current HE students.
  MePlusMe can be accessed on a computer or mobile device. Users can follow two routes to receive a customised package suited to their specific needs: a symptoms-route (Questionnaire) or a techniques-route (Library). The questionnaire statements were developed from validated questionnaires widely used in clinical settings [Hospital Anxiety and Depression Scale (HADS); 7-item Generalised Anxiety Disorder Scale (GAD-7); Patient Health Questionnaire (PHQ-9)] and a formal interview [Mini International Neuropsychiatric Interview (MINI)]. The results of this assessment inform the selection of techniques to address the symptoms faced by the user. Alternatively, the users can assemble their own packages with the help of an online ‘library’. Students can access and practice the techniques in their package in their own space and time, and over time use a rating system to monitor their progress. Throughout this process, MePlusMe provides optional support via emails to encourage continued use and engagement.
  The techniques used in MePlusMe are based on CBT, mindfulness, and educational practices, presented in an animated video format using either a male or a female character. Although the system is aimed at students with mild to moderate mental health and academic difficulties, it could also be used by the general student population. As well as individual and personalised packages, the system offers a built-in moderated social network, “Thoughtwall”, which aims to enhance the feeling of belonging to a student community with similar difficulties.
  MePlusMe is still in development, and research to support the system is ongoing. Initially, a small survey of 61 students was conducted to determine their opinions of online support systems (Goozee et al. submitted). This indicated that the majority of students would use such a system. They also specified that it should be interactive and personal, with techniques to support time and money management, relaxation, and social skills. A proof-of-concept study was subsequently conducted, to support the conceptual and practical value of the system among students at five UK HEIs [26]. Students were largely positive in response to the system, but the study highlighted areas for improvement, which were incorporated into the subsequent development of the system.
  A feasibility study will commence in September 2017. The study will evaluate the feasibility, acceptability, and potential effects of the online tailored interventions offered in MePlusMe to address mild to moderate psychological and study skills difficulties in HE students, as well as its content. For the purpose of the study, 200 students will be recruited to a within-subjects, repeated measures study conducted over 8 weeks. A more in-depth feasibility study [27] and a subsequent randomised controlled trial (RCT) are then planned.
- *Mindful Kiwi *(http://www.mindful-kiwi.com) Mindful Kiwi offers an 8-week programme, based on techniques from Mindfulness-Based Stress Reduction (MBSR) that are adapted to focus on the needs of HE students and healthcare professionals. There are two programs: (i) Mindfulness-Based Coping with University Life, which aims to support university students, and (ii) Mindfulness for Therapists, which was designed for therapists and other healthcare professionals.
  Mindfulness-Based Coping with University Life focuses on four areas: managing stress, academic performance, health and wellbeing, and communication and relationships. Mindfulness for Therapists aims to improve healthcare professionals’ wellbeing. The training is delivered either face-to-face or online, synchronously and asynchronously. Both versions provide access to a group forum and users of the synchronous delivery option can also communicate with a group facilitator. One study has investigated the approach used by Mindful Kiwi [28]. The study was a non-randomised pilot evaluation, using a waiting list as a control group, in students from a UK HEI. Ten students used Mindful Kiwi across eight weeks, while six controls were placed on a waiting list. The results suggested that the programme is easy to implement and that students receiving the active intervention showed reductions in negative mood and anxiety, but no changes in biological indicators of stress (cortisol). However, there were no significant intergroup differences at follow-up in measures of anxiety, depression, or perceived stress.
- *PLUS* (*not yet live*) Personality and Living of University Students (PLUS) was designed by researchers at King’s College London and aims to tackle symptoms of anxiety and depression, and to improve self-esteem of HE students. Interventions target personality traits known to be risk factors for common mental disorders, namely Neuroticism, Concern over Mistakes, Doubts about Actions, and Hopelessness. Techniques are based on CBT and are presented as text-based exercises. Users are first guided through nine questionnaires to identify their strengths and weaknesses, with assessment of alcohol and drug use, eating disorders, self-esteem, personality traits, quality of life, depression, anxiety, and phobias. Following a general introduction to the principles of CBT, PLUS then offers the following modules to support the student: ‘anxiety, worry and panic’, ‘dealing with difficult emotions’, ‘self-esteem’, and ‘perfectionism’. These can be completed in any order, and consist of text-based information with photographs and illustrations.
  PLUS has been investigated in a randomized controlled trial of 1047 students from two London universities [10]. The personality traits of students were assessed at baseline and the students were divided into groups of high and low risk for mental disorders. They were then randomised to receive the online trait-focused CBT (PLUS) or a control intervention (online information about accommodation, finances, and study skills). Anxiety and depression (PHQ9 and GAD7), alongside secondary outcomes, were assessed at baseline, 6 and 12 weeks. There was a high attrition rate, with 401 students completing the 12 weeks’ assessments. However, high-risk students were not more likely to drop out and the dropout rate (61.7%) was not greatly higher than for other web interventions. The authors reported decreased anxiety and depression at 12 weeks in high-risk students completing PLUS. They also assessed user satisfaction, with students reporting that the site was easy to use, they liked the design, and they found reminders useful.
- *Students Against Depression* (studentsagainstdepression.org) Students Against Depression is a website offering information and advice about depression, anxiety, and suicidal thoughts for students in higher education. The website was established by and is now run by Student Minds (a student-led UK mental health charity) working with the Charlie Waller Memorial Trust. Students Against Depression provides information about the mental health problems it addresses, as well as tools and strategies for self-help, such as planning a daily routine. These techniques are theoretically grounded in CBT. Users can also complete a blog and share their story.
  The website does not offer users access to clinicians, but does encourage students to contact counsellors and doctors. In the autumn of 2014, Student Minds and Students Against Depression collaborated to produce Positive Minds, a 6-week offline course designed to give students the skills they need to fight depression and anxiety. The Positive Minds course launched on three campuses in the UK in 2014. Since then it has been re-launched and expanded to encompass eight campuses in 2016, including Bath, Bath Spa, Bournemouth, KCL, Nottingham, Oxford, Oxford Brookes, and Southampton. The course is run by student facilitators trained by Student Minds. The site has not yet been subjected to empirical research.
- *theDesk *(http://www.thedesk.org.au) TheDesk is an Australian support system designed for HE students, but can be used by anyone registered with the website. The system provides help for various issues, from academic study skills to depression, substance abuse, and issues with sexual orientation.
  After a self-assessment of wellbeing through quizzes, the website recommends one (or more) of four different modules (Getting things done, Staying calm, Staying connected, and Feeling good). Each module contains videos and tips for effective time management, management of emotions, tips to avoid procrastination, breaking down problems, etc. Throughout, the users are asked to list and evaluate their own strategies for coping with different problems, as well as to assess themselves.
  If the results of the self-assessment suggest that professional help is needed, the website redirects the user to a Get Help link, providing professional helplines. For emergency situations (suicidal thoughts or self-harm), a suicide prevention helpline is listed. The website also provides recommendations of several apps that can increase wellbeing. The site has not yet been subjected to empirical research.

#### (ii) Systems designed for the general public or other groups

In this section, we attempt to describe similar systems that have been designed for the general public or other groups, but may nonetheless be of some usefulness to student populations.

- *Beating the Blues *(http://www.beatingtheblues.co.uk) Beating the Blues is the mental health support system most widely used by the UK National Health Service (NHS), available to the general public. It is available as a paid-for service or for free with a prescription. It provides an 8-week course of CCBT for anxiety and depression. Participants learn about the causes of their psychological difficulties and how to overcome them, setting personal goals to tailor the programme to their individual needs. They work through a series of narrated screens, which provide psychoeducation and CBT techniques, alongside case studies as examples and interactive questions.
  Beating the Blues was appraised by the National Institute for Health and Care Excellence (NICE) in 2006, and subsequently recommended for people with mild or moderate depression in both primary and secondary care. NICE now recommend CCBT as a whole without specifying a particular programme. However, Beating the Blues is the system with the largest evidence base supporting it, with 10 studies conducted, covering its initial development and its effectiveness in various populations [29–37], including a study of a HE student population [38].
  Proudfoot et al. [29] described the initial development of the system and reported positive results (patient-reported usefulness and improvements on clinical measures) from a beta-test of 11 patients. Two subsequent RCTs were conducted [30, 31], which compared patients with anxiety or depression who were randomly allocated to receive treatment as usual or support from Beating the Blues. In both RCTs, benefits were seen for patients receiving online support from Beating the Blues, with associated increases in work and social functioning. A later naturalistic open trial showed improvements in clinical and occupational measures for 219 patients, supporting the effectiveness of the system within routine care [32]. A cost-effectiveness study of the system’s use in GP settings reported that Beating the Blues was both more expensive and more effective than treatment as usual [33]. However, only the difference in effectiveness was significant and so the authors suggested that the substantial treatment benefit for a non-significantly greater cost offered cost-effectiveness. Further studies have been conducted reporting the system’s effectiveness in treating anxiety and depression within primary care [34], a community mental health team [35], and specialist CBT centres, including inpatients with physical comorbidities [36, 37].
  As mentioned above, and relevant to this review, one study applied this system to a group of higher education students [38]. A small sample of 12 depressed students used the programme, with only 10 completing it. The study showed significant reductions in depression, but no changes in anxiety. Finally, participants were very positive about the system, in some cases preferring it to face-to-face counselling.
- *Big White Wall *(http://www.bigwhitewall.com) Big White Wall is a UK-based digital service provider designed for the general population that can either be accessed through NHS referral or through a private subscription. Its primary goal is to provide early intervention and to determine the need for further mental healthcare. It is also dedicated to providing 24/7 support for users to address issues before, during, and after therapeutic interventions. The services can be accessed through the website or through an app for mobile devices. The app is listed in the NHS health apps library.
  Big White Wall uses internet-based CBT and peer support (anonymous support groups guided by trained professionals) to tackle mental health difficulties, including anxiety, stress, and depression. Furthermore, Big White Wall offers self-assessment and information about various topics, such as abuse, bullying, relationships, and lifestyle. It also encourages self-expression in the form of a ‘Brick’ that members create to express their emotions. These ‘Bricks’ are shared and the underlying stories are discussed in the support groups. Big White Wall also offers synchronous one-to-one therapy with a clinician through the digital platform.
  An independent review has been conducted to evaluate the effectiveness of Big White Wall’s digital services [39]. The review was based on a survey of 598 active users and had a response rate of 38.3%. Overall, the respondents reported reduced isolation (45%), reduced depression (40%), and increased self-understanding (52%) after using Big White Wall. Three factors appeared to be essential in the decision to use Big White Wall: connection with others struggling with the same difficulties, lack of alternatives, and ease of access. At the time of the independent review, the majority of Big White Wall users were female (80%), aged 16–24 (50%), and from urban settings (80%). Subsequently, it was estimated that Big White Wall compared with traditional therapy created savings to mental healthcare costs of £36,935 per 100 members [40].
  Dosani et al. [41] published their observations of the use of Big White Wall in Wandsworth, London. They noted that support from others with similar experiences through online support networks and access to online therapy with flexible times improves coping and adaption skills of users, and changes their mental health for the better. However, several authors reviewing online systems, including Big White Wall, have highlighted the concerns for safety and how to manage crises in those accessing online support [40, 42], citing the need for moderation by professionals.
- *e-couch* (ecouch.anu.edu.au) Designed for the general population, e-couch is an Australian self-help and support program developed by the Australian National University (ANU). It is also a research enterprise that gathers data about issues linked to depression and anxiety. The system provides self-help strategies and a research platform to educate users about coping strategies for depression and anxiety. It provides advice on relaxation and physical activities, a self-help toolkit, and an internet support group.
  An initial small RCT was conducted comparing three groups (*n* = 21) with generalized anxiety disorder (GAD) who were randomized to receive e-couch (active website), the selective serotonin reuptake inhibitor (SSRI) sertraline, or a control website for 10 weeks [43]. The participants were compared post-test and at 6 and 12 months follow up on a measure of anxiety (GAD-7 scale) and depression (CES-D). The authors reported decreased depression symptoms in those randomised to e-couch or the SSRI, but not the control website. The active website also showed benefits in treating GAD compared with the control website at immediate post-test, but these benefits were not maintained at longer-term follow up. There also appear to be secondary benefits from the use of e-couch compared with a control intervention, with improved self-esteem and empowerment immediately after use [44]. Participants who used e-couch also had higher perceived quality of life 6 months after use compared with the control group.
  Factors related to the effectiveness of e-couch have been explored in several studies, which have shown that the number of activities per log into the site is associated with a clinically significant outcome [45]. Furthermore, bigger reductions in depression scores at follow up have been reported for women and those with lower levels of dysfunctional thinking at baseline [46].
  Studies have also investigated the user’s perspective of e-couch. An online survey of 298 users suggested that participants felt online interventions could improve understanding of depression, but were less confident that it could provide skills for preventing it [47]. A qualitative analysis of e-couch was conducted with 12 participants who had comorbid depression and cardiovascular risk symptoms [48]. Semi-structured face-to-face (*n* = 2) or telephone (*n* = 10) interviews were analysed using a grounded theory approach, revealing barriers to and motivations for use of the system. Barriers included time constraints and limited perceived worth of the system, while motivations for use included perceived improvements and a sense of control. The same authors subsequently conducted a randomised, double-blind trial in a much larger sample (*n* = 562) from the same patient population, comparing e-couch with a health information website control [49]. Intention to treat analyses suggested that those receiving 12 weeks of e-couch showed bigger reductions in depression symptoms (measured using the PHQ-9) than those receiving the control intervention, with the difference between the groups increasing over time.
- *FearFighter™* (fearfighter.cbtprogram.com) Developed by CCBT Ltd. for the general population, FearFighter™ supports users with generalized anxiety, panic, and phobias. They receive CBT over a period of 9 weekly sessions, each lasting approximately 50 min. The system has been approved by NICE, and is available within the NHS and health services in other countries. Although patients can access the system via their healthcare professional, it can also be purchased privately.
  A variety of research studies have been conducted to test the clinical and cost effectiveness of FearFighter™, including RCTs. Initial pilot and feasibility studies suggested that FearFighter™ could lead to similar improvements for patients with phobia or panic as clinician-guided treatment with a reduction in clinician time [50, 51]. Another initial study in which participants used FearFighter™ at least six times over 12 weeks, and were supported by brief telephone or face-to-face contact with a therapist, found improvements in 41% of patients using the system [52]. The authors stress that these systems could be used as part of a ‘stepped care’ system, in which patients are initially provided computer self-help and then are stepped up to face-to-face therapist support where appropriate.
  To date, two RCTs have been conducted. In the first RCT, 93 patients with panic or phobia were randomised to self-exposure therapy via Fearfighter™ or face-to-face with a clinician, or to computer-guided relaxation [53]. They reported no significant differences between the two groups undergoing self-exposure, suggesting that computer-guided therapy is as effective as face-to-face therapy. There was a higher rate of dropout for computer therapy, but also 73% less clinician time per patient. It is possible therefore that computer therapy allows for equal effectiveness, but at a lower cost. Indeed, a preliminary economic analysis by the same authors suggests that Fearfighter™ is more cost-effective than face-to-face therapy [54], although they stress that this is a secondary analysis of data and so should be interpreted cautiously. Following a feasibility study in rural regions of Scotland that suggested general satisfaction and perceived improvement of the participants [55], an RCT was conducted in the same regions [56]. Participants referred by their GP or self-referred were over 16 years old with phobic anxiety or panic, and had 10 weeks of unlimited access to the system. Twenty-six patients completed the CBT sessions and 19 were assessed at final follow up, showing significant improvements in symptoms. While this is promising, no comparison intervention was delivered to a control group.
- *Living Life to the Full *(http://www.llttf.com) Living Life to the Full (LLTTF) is available to the general public and was developed by a psychiatrist in the UK. LLTTF aims to improve its users’ coping with mental health issues, including low mood or depression, anxiety, worry and panic, phobias, obsessive–compulsive disorder (OCD), and bulimia. It uses CCBT techniques, as well as self-help and group support in the form of online forums. It also offers the possibility to connect with one’s own practitioner online, so that information such as depression scores, notes, and messages can be accessed. Furthermore, advice and support is offered for physical wellbeing, for example in the areas of diet and exercise. The site has not been subject to empirical research.
- *MoodGYM* (*moodgym.anu.edu.au*) MoodGYM is a free internet-based training program designed to help people cope with troubling, but not incapacitating, symptoms of depression. It was developed in Australia, and has since been translated into Norwegian, Chinese, Dutch, and Finnish. It was designed as a prevention program for young people aged 15–25 years old, but users of all ages can register. MoodGYM primarily aims to help people experiencing depressive symptoms. It includes five modules that teach users to cope in different situations: ‘Feelings’, ‘Thoughts’, ‘Unwarping’, ‘De-stressing’, and ‘Relationships’. Users complete self-assessment quizzes, which may lead to a referral to a GP or therapist. The therapy is based on CBT and interpersonal therapy, and is presented with animated demonstrations, quizzes, and “homework” exercises. MoodGYM has an emergency page with information for people feeling suicidal, and people who know someone feeling suicidal. It lists a number of helplines with phone numbers and it encourages people to speak out.
  A large number of studies have investigated MoodGYM across a range of situations and using various study designs. Studies have reported decreased depression after use of MoodGYM in patients with traumatic brain injury [57], in adolescent girls in a school setting [58], and in patients attending general practices in Australia [59]. In a study of use of the site over 6 months, Christensen et al. [60] found that people accessing MoodGYM had baseline levels of anxiety and depression that were greater than in the general population, and that these symptoms decreased significantly after completion of modules within the system. However, Twomey et al. [61] found that there were no significant improvements in depression and anxiety symptoms in a group of mental health users compared with a waiting list control, although general psychological distress did decrease. They suggested CCBT may be a suitable adjunctive treatment rather than a first-line intervention.
  In a qualitative study, 14 patients with depression were asked about their experiences of using MoodGYM [62]. They reported that it made them feel as though they were doing something about their problem, providing knowledge to apply to their difficulties. But they highlighted the importance of therapist interaction, and that a trusted relationship with a professional is fundamental.
  Several RCTs have investigated the effectiveness of MoodGYM, often compared with waiting list controls or psychoeducational websites. Some of these studies reported improvements in symptoms of depression. In one study of female secondary school students (*n* = 157), the use of MoodGYM was compared with the school’s standard personal development activities. The effect size for the reduction in depressive symptoms following the use of MoodGYM was not significant immediately following the intervention. However, 20 weeks later the effect size was moderate and significant [58]. Another RCT of the same system in primary care patients with depression found improved measures of recovery [63]. It should be noted that in the latter study, the intervention was therapist led, and included face-to-face support and email communication. Yet another RCT found a reduction in symptoms of depression in users of MoodGYM similar to that of users of a psychoeducational site, and greater than the control intervention (attention placebo) [64]. However, others have reported no benefit when secondary outcomes were examined, perhaps due to low uptake [65] or when compared with the use of informational websites [66].
  It is possible that response to CCBT shows individual variability, and reported predictors of good response include more episodes, being married or living with a partner, and high life satisfaction [67]. Schneider et al. [67] investigated acceptability of MoodGYM compared with information websites within an RCT setting. They found that there was high acceptability over time, including when compared with face-to-face therapy, with 60% of surveyed participants holding this view. Nonetheless, several barriers to use were identified, including personal views of CCBT at baseline, lack of time, technical issues (e.g., page keeps refreshing), and the fact that it is too impersonal. As can be seen from this, results have been mixed. Indeed, a meta-analysis has shown only a small effect of MoodGYM on reducing depression symptoms that became non-significant following adjustment for publication bias [69]. The authors suggest that the control group, the level of clinician guidance, and adherence may all confound the results of RCTs. A study comparing the outcomes of participants in an RCT with spontaneous users of MoodGYM suggested that there were no differences in user demographics (such as age and gender) or the improvements seen, although RCT participants did complete more assessments [70].
  Several studies have investigated adherence, which is an important factor given that greater adherence may be related to better outcomes [71]. This suggests engagement and adherence are key if target populations are to benefit from CCBT. Lillevoll et al. [72] looked at engagement with MoodGYM in a school, reporteing a low level of uptake (8.5%) and little use beyond the first few pages of the website. They therefore suggest that communication and psychoeducation about the benefits of such programmes may be necessary to encourage use. Another school-based study compared adherence to MoodGYM in those accessing the site in a monitored classroom-based setting with spontaneous users, finding greater adherence in the former [73]. This highlights that even if effective in treating depression and anxiety, developers of such systems must also consider how they engage users and the settings in which they are likely to use them.
  Other outcomes have also been investigated. Griffiths et al. [22] investigated the effects of MoodGYM and an informational website on stigma towards depression, finding reduced personal stigma following both, but an increase in perceived stigma following MoodGYM. The effects that stigma may have on effectiveness and use of such systems will need to be carefully considered.
- *NHS Silvercloud* (uk.silvercloudhealth.com) NHS Silvercloud was developed by a team of both clinicians and researchers. It is a platform that hosts treatment programmes for several disorders, and is available to NHS patients. It can support people with depression, social anxiety, health anxiety, generalised anxiety disorder, eating issues, and obsessive compulsive disorder, and it also collects data for research from users. It is available by referral only to those over 16 years old. Silvercloud is a platform through which various different interventions, mostly based on CBT, can be delivered depending on the presenting problem.
  Each user has a personal space in which they can provide details of their problems, document their thoughts and experiences, and from which they access their modules. The interventions are delivered as interactive modules and users can provide feedback and comments on the content. They are supported asynchronously, with weekly reviews from their supporter, who can receive messages but also completed exercises and comments from the user. Limited, moderated interaction between users is also possible, in an anonymised form. Various interventions are available for different disorders, such as depression (Space from Depression, MindBalance), anxiety (Space from Anxiety), and stress (Space from Stress).
  There has been some research conducted to assess the individual interventions offered by NHS Silvercloud. An initial exploratory study of the MindBalance programme was undertaken within an Irish university counselling service [74]. Twenty-eight first and second year undergraduates were recruited by email, but only 18 completed both the pre- and post-intervention assessments. A significant decrease in depression symptoms (measured with the BDI) was observed, with a much lower dropout rate that has been reported elsewhere (36%). It is possible that the presence of a supporter throughout use of the system may improve adherence. A similar study of 80 students using MindBalance at Trinity College, Dublin, reported a statistically significant reduction in depression scores, with 74% of participants in a lower BDI category at the end of the study [75]. Those with more severe depression also spent more time using the system than those with mild or moderate depression. Severity of symptoms may thus be a factor in dropout from studies or CCBT programs, as those with milder symptoms may find a shorter period of intervention effective and so no longer feel compelled to continue use of the system.
  The Space from Depression program has also been evaluated in two RCTs [76, 77]. Participants were recruited through the Aware mental health charity and were mainly female, highly educated, and in full time work or study. In the first trial, 188 participants were studied, 96 of who were randomised to the treatment and 92 were put on a waiting list control [76]. Only 36 of those receiving treatment completed all modules, but the authors suggested that the non-linear nature of the intervention means there is no need for every individual to complete all modules in order. In fact, such flexibility may be a motivator for better engagement. Nonetheless, there were significant decreases in both depression and anxiety symptoms, which appeared to be maintained at 3 and 6 month follow up. The second trial included 281 participants from the same sample and investigated users’ satisfaction with the program [77]. Participants found it easy to use and were happy to use a computer to access treatment, and liked the flexibility and accessibility of the program. They also found the presence of an online supporter helpful and motivating.
  One study reports on the engagement of Irish students with the anxiety program offered by NHS Silvercloud [78]. They recruited seven students who had participated in a trial of the system to take part in semi-structured interviews. They identified several motivators for engagement, such as being able to see progress made, flexibility, and the online supporter, whereas identified barriers included other priorities, and problems with the layout and navigation through the system. Importantly, users reported that the social functions of the system made them feel less isolated and that they were not alone in experiencing anxiety.
- *ReachOut * (http://www.reachout.com) ReachOut was the first online youth mental health service, which was developed by the Inspire Foundation in Australia in 1996. Focusing on young people aged 12–25, it aimed to increase mental health literacy and promote resilience, social connectedness, and help-seeking behaviours [79]. The system has subsequently begun to operate in the United States and Ireland. It provides young people with information and support across various areas of mental health issues, such as depression, anxiety, eating disorders, substance abuse, self-harm, and suicidal thoughts. Additional topics addressed include loss and grief, bullying, LGBTQ identity issues, family relationships, and study skills. This is all provided in the form of factsheets, tools, apps, and blogs, alongside peer support in community forums. Users can post questions or comment under a relevant section (e.g., the Study Skills Factsheet) and a ReachOut moderator will reply within 24 h. Although counselling is not offered, ReachOut signposts young people to professionals who can help in times of crisis and encourages them to speak out.
  Several extensions to the programme have subsequently been developed, including ReachOut Professional to help youth workers, health professionals, and schools support the mental health and wellbeing of young people. ReachOut 101 is a short online course for people who want to increase their mental health literacy. It aims to improve understanding of issues such as where to find mental health information online, how to access online mental support, and how to communicate in an effective and polite way in an online community (so-called *netiquette*). Further extensions include ReachOut Forums (to reduce stigma and promote help-seeking) and ReachOut TXT (a peer-to-peer mobile service providing teens and young adults confidential support and information by text message).
  ReachOut has conducted annual user surveys (all available via their website: http://about.au.reachout.com/us/our-research/) to explore the demographics of visitors, their help-seeking knowledge, preferences, and behaviours, as well as their attitudes to mental health. ReachOut also conducts an annual commentary analysis, to understand the types of conversations and topics visitors are interested in. Burns et al. [80] reported on one such user survey from 2008. They considered the implications of online support given that 90% of young people use the internet while very few are help-seeking for mental health problems. In common with other research investigating ReachOut use, the majority of respondents were female. Of the respondents, 87% had used the site previously, and they reported that the site was trustworthy and there when they needed it. The authors further reported that the site encouraged further help-seeking in 59% who talked to a professional following use of the site. The most recent user survey available is from 2013, and reported on use by 2600 visitors to the site. Seventy-seven percent of respondents reported high levels of psychological distress, and half of first time users had not accessed other professional help before using the system. The authors reported general user satisfaction with ReachOut and that 81% of users would return in the future to the site.
  An early investigation of ReachOut surveyed 243 students aged 13–18 years (51% male) in schools across Australia [81]. Students attended a presentation about help-seeking that focused on ReachOut and 6 months later completed a questionnaire assessing help-seeking knowledge, intentions and behaviour, and whether they would use ReachOut during a time of crisis. They found that the majority of students knew where to seek help but their intention to seek help would depend on the problem. There was a gender difference in how likely students were to seek help, with male students more likely than female students to report that they would keep their problems to themselves. The same research group later presented ReachOut as a case study for online mental health interventions [82]. They reported over 7 million visits to the site since it was launched and suggested that a third of young Australians had heard of the site.
  In another programme evaluation, Collin et al. [79] considered the extent to which ReachOut encourages help-seeking among young people. Assessing website statistics (via Google analytics) and the results of a cross-sectional survey of users, they reported a high number of visitors to the site, with pages on depression and mood disorders particularly popular. They found that the majority of users were 14–25 years old and female, and 32.5% of survey respondents reported that they had sought help from a professional following use of the system. However, the majority would rather seek help from family or friends than from a mental health service. This may be seen as a failure of the system to increase knowledge and help-seeking among this population, but it might also highlight the importance of online support for those who are unlikely to seek help elsewhere. It might also be important to identify the reasons why people might not wish to seek help from professionals.
  Recognising the important role of parents in the mental health of young people, ReachOut developed a programme called Bridging the Digital Disconnect, which aimed to develop online resources for adults, including parents [83]. As part of this, parents’ views of the content, appearance, and appeal of the site were investigated. Twenty-one parents rated sections of ReachOut on 10-point Likert scales and all ratings were consistently high (> 8). However, they identified some areas for improvement, including the ‘Ask the Expert’ videos, which they deemed too theoretical and drab, and certain content that they thought may be distressing.
  Finally, a number of apps and games have been developed by ReachOut, aimed to increase engagement with particular groups. For example, ReachOut Central, was a game developed to increase engagement of young people. Burns et al. [84] reported that there was good uptake of this game and that it led to a reduction in psychological stress for users, but this was only seen in women and not men. They suggested that research is required to identify ways to keep young men engaged in apps or websites that support mental health.
  ReachOut continue to conduct research projects about topics ranging from cost reduction in mental health care to a cohort study for online healthcare evaluation. They also develop apps and online wellbeing games to further enhance young people’s experiences. Further information about these ongoing projects can be found at their website.
- *SuperBetter* (superbetter.com) Created by game designers, SuperBetter is an online tool and gaming system that helps users build personal resilience, and to boost their physical and mental wellbeing. It is aimed at the general population, but university educators can incorporate the game into their coursework for students.
  SuperBetter aims to help users beat depression, overcome anxiety, cope with chronic illness or chronic pain, heal from physical injury, or recover from post-traumatic stress. Additionally, it promises to support users to improve their lifestyle, overcome challenges, and complete projects in a focused manner. It follows a game-like programme with achievement targets set at the beginning by the user and progress tracked over time. Users have to complete ‘quests’, activate ‘PowerUps’, and battle ‘Bad Guys’. It can serve as a self-management tool, and progress can also be tracked and encouraged by others. It also provides the option to play with other users with similar experiences (‘Allies’).
  One study has reported that playing SuperBetter for 30 days significantly reduces symptoms of depression and anxiety [85]. Participants in the study were self-selected and recruited online, so likely to be highly motivated. Despite this, there was a high dropout rate (only 18.34% of the sample remained at follow up) and as such, the results should be interpreted cautiously. The participants also only met criteria for subthreshold depressive symptoms, limiting the generalisability of the results to those with clinical levels of psychopathology. Furthermore, no RCTs have yet been conducted and so it is difficult to assess effectiveness at this stage.
- *UBalancer*(ubalancer.com.au) UBalancer is a coaching program, developed in Australia, that can be customised for educational purposes. It targets the general population, but the software can be used in a number of areas, such as education, sport, or business. The system combines a cloud-based software program with coaching, via one-on-one meetings or interactive group workshops. It promises to help users who are feeling overwhelmed or suffering from stress or anxiety. It also provides coaching in sleep and time management, as well as an introduction to mindfulness. The coaching sessions last 60 min and are offered via Skype. The site has not been subjected to empirical research.

## Discussion

Following the recent proliferation of web-based mental health and wellbeing support systems available to HE students, a review of online support systems is timely. It is also important to assess the research evidence supporting these systems. While some systems have not yet been subjected to scientific scrutiny, many have a number of studies investigating their use and effectiveness. For the purposes of the present review, the Oxford Centre for Evidence Based Medicine Levels was used to rate the effectiveness (treatment benefits) of each intervention [25]. These are outlined in Table 2.

All interventions achieve a Level 5, in that they have been built according to mechanism-based reasoning, usually based on CBT or occasionally mindfulness-based techniques. Moreover, some of the interventions have case studies supporting them, achieving a Level 4. A few of the interventions have extensive empirical evidence, in the form of non-randomised studies or very small numbers in RCTs (Level 3). Only one of the systems designed specifically with HE students in mind (PLUS) has a substantially big RCT as evidence, achieving a Level 2. In contrast, of the systems that were not specifically developed for HE students, three achieved a Level 2, with more than two RCTs assessing their efficacy. It is important to note that there are no systematic reviews of RCTs for any of the systems reviewed.

Although relatively new, some interventions have reported promising results in reducing mental health difficulties, such as anxiety and depression. They have also reported improved mental health literacy and reduced stigma. There are significant similarities between some systems, but some distinctions can also be drawn. Some of these are outlined below.

### Mode of delivery

The systems identified in this review can be divided into three broad categories according to the way in which the intervention is delivered (although some systems do use more than one mode of delivery in parallel):

- *Therapist*-*guided treatment using computer*-*based cognitive behaviour therapy* For some systems, web-based therapy is delivered under the guidance of a therapist (Big White Wall, NHS Silvercloud), which may be synchronous or asynchronous. Others offer one-on-one therapy through a computer application such as Skype (UBalancer). However, there are no detailed reports of the availability, duration, cost, or functionality of these systems. Furthermore, almost all systems offer the opportunity to track progress via online journals, blogs, self-assessment questionnaires, or emails. Finally, CBT can be regarded as a primary treatment intervention or as supplementary to work with a therapist, but no studies provided robust evidence that either of these models is preferable.
- *Self*-*help systems based on CBT techniques without direct involvement of a therapist* Some systems offer self-help without therapist guidance, providing tools and techniques for users to implement independently (Beating the Blues, CALM, e-couch, MePlusMe, Mindful Kiwi, MoodGym, PLUS, ReachOut.com, Students Against Depression, Superbetter.com, theDesk, UBalancer). However, the only system that offers a personalized intervention depending on individual needs is MePlusMe because it addresses symptoms as opposed to diagnosed conditions, while the other systems offer the same pre-made packages or modules to all users for specific conditions. As a consequence, the packages MePlusMe offers vary each time depending on users’ current difficulties. As such, MePlusMe facilitates multiple uses from same user, which can positively affect recurrent engagement as it is solution-focused, brief, and tailored for each visit. Many such systems offer peer support, where users can communicate anonymously using a ‘sharing wall’ or online forum (Big White Wall, ReachOut.com, Students Against Depression, MePlusMe). These forums are sometimes supervised by professionals, but support often only consists of automated reminders.
- *Educational and research platforms for mental health* In addition to offering support for wellbeing, web-based systems often also serve as an educational and research platform for mental health issues (e-couch, NHS Silvercloud, ReachOut.com). All offer those suffering from mental disorders, as well as family and friends, can retrieve information about the difficulties they are experiencing, as well as coping strategies. MePlusMe also offers psychoeducational videos to help users understand their experiences. Systems that do not offer real-life support have a ‘panic button’ (Living Life to the Full, MoodGym, ReachOut.com, Students Against Depression, theDesk, MePlusMe). This takes the user to advice about who to contact immediately if they are feeling suicidal or desperate.

### Problems addressed and target group

The systems offer support for a range of potential difficulties. All systems reviewed offer help for stress, depression, and anxiety. Some systems provide additional support for substance abuse (CALM, PLUS, ReachOut.com), LGBTQ-related concerns (ReachOut.com), relationship difficulties (e-couch, Mindful Kiwi, theDesk), general wellbeing and lifestyle choices (Big White Wall, MePlusMe, Mindful Kiwi, Superbetter.com, theDesk), suicide and self-harm (ReachOut.com, Students Against Depression), and eating disorders (NHS Silvercloud, PLUS). Beating the Blues and MePlusMe are the only systems targeting sub-clinical problems, such as mild to moderate depression symptoms although the former also offers support for severe cases. In contrast, MePlusMe is based on a continuum model of mental health. As such, its content is presented in a way that normalises certain difficulties and can thus be adopted by users who have no symptoms but wish to improve their personal effectiveness.

Most web-based systems addressing mental health and wellbeing were designed for the general population (Beating the Blues, Big White Wall, Superbetter.com, UBalancer) although these are also available for students to access. However, several systems have been designed for specific groups, targeting young people (MoodGym, ReachOut.com) or students specifically (CALM, MePlusMe, PLUS, Students Against Depression, Mindful Kiwi, theDesk). Furthermore, some also offer study skills support (e.g. MePlusMe, Mindful Kiwi, ReachOut.com, Students Against Depression, theDesk).

### Evidence for effectiveness

There has been a proliferation of web-based mental health support systems in recent years, and some research exists to support the effectiveness of some online self-help tools in the general population (e.g., see [20]). However, not all available systems have been evaluated to date. Table 2 provides a useful overview of the evidence available for each system.

Systems that are proposed for use within university counselling services must be supported by studies of their effectiveness. If such studies support their effectiveness, as well as acceptability among the targeted populations, they will likely provide a cost-effective way to meet the needs of students with mental health difficulties, easing the financial burden of mental health care on university counselling services [86].

If web-based interventions are to be useful, then ensuring engagement and adherence among users is a particular area of concern. Various studies have investigated the motivators and barriers to engagement. Often, flexibility and being able to track progress are reported as factors that can keep a user motivated to complete modules. The use of an online supporter who reviews your progress has also been reported as helpful. Understanding the barriers to use or to continued engagement will be key to ensuring that online systems are successful. Often users cite competing priorities as a barrier. As such, systems can increase their acceptability if they are easy to use, able to be accessed on the go, and offer immediate, relevant, short, and quick solutions rather than lengthy, inflexible, and rigorous modules to complete. Ensuring that people are aware of the help available to them and the potential benefits of web-based systems may also increase engagement.

### Online support systems in HE

Students in HE can benefit from the opportunities offered by web-based interventions for anonymous and non-stigmatizing access to tools that support mental health and wellbeing. Furthermore, such systems could aid improvements in academic skills, by offering support for study and time management skills. Online support can offer benefits to universities and other HE institutions by optimizing their resources, improving retention rates, and minimizing the cost of drop out from courses. For example, such web-based interventions could be offered to students with mild to moderate levels of depressive symptoms, while counselling services focus on those with more severe problems that require more intensive interventions. Higher education institutions can also meet their pastoral duties in a more effective way by offering a solution that is accessible, personal, and can reach out to all students. Higher education institutions often still primarily rely on traditional platforms to provide student support services. This fails to capitalize on the advantages offered by web-based systems, such as allowing students to benefit from a support environment that facilitates their own learning activities, as well as self-managed activities to improve their wellbeing while maintaining connections to their university, peers, and support networks across time and place. However, more research is required to support the effectiveness of such systems, as well as to identify the best modes of delivery, how to keep users engaged, and how to reduce barriers to use.

### Conclusion/future directions

In this paper, we provide a review of support systems available to HE students facing mental health, wellbeing, and study skills difficulties. A large number of such systems are now available, and while the evidence so far suggests that they could be useful and cost-effective it remains to be seen whether they will provide a viable alternative to human, face-to-face support.

The research already conducted and outlined in this paper is largely positive, but larger RCTs are still needed to provide more robust evidence. Results from such studies could support the use of web-based alternatives in HE institutions, as an independent or complementary solution to campus mental health services, to improve student mental health and wellbeing and relieve the burden on overstretched student support services.
